# Monoamine Oxidase B Expression Correlates with a Poor Prognosis in Colorectal Cancer Patients and Is Significantly Associated with Epithelial-to-Mesenchymal Transition-Related Gene Signatures

**DOI:** 10.3390/ijms21082813

**Published:** 2020-04-17

**Authors:** Yi-Chieh Yang, Ming-Hsien Chien, Tsung-Ching Lai, Chia-Yi Su, Yi-Hua Jan, Michael Hsiao, Chi-Long Chen

**Affiliations:** 1 Graduate Institute of Clinical Medicine, College of Medicine, Taipei Medical University, Taipei 110, Taiwan; quaint29@gmail.com (Y.-C.Y.); mhchien1976@gmail.com (M.-H.C.); 2 Department of Medical Research, Tungs’ Taichung Metro Harbor Hospital, Taichung 433, Taiwan; 3 Genomics Research Center, Academia Sinica, Taipei 115, Taiwan; chuching0305@gmail.com (T.-C.L.); joysuforlab@gmail.com (C.-Y.S.); isaacjan@gmail.com (Y.-H.J.); 4 Pulmonary Research Center, Wan Fang Hospital, Taipei Medical University, Taipei 116, Taiwan; 5 Traditional Herbal Medicine Research Center, Taipei Medical University Hospital, Taipei 110, Taiwan; 6 Division of Pulmonary Medicine, Department of Internal Medicine, Wan Fang Hospital, Taipei Medical University, Taipei 116, Taiwan; 7 Department of Biochemistry, College of Medicine, Kaohsiung Medical University, Kaohsiung 807, Taiwan; 8 Graduate Institute of Cancer Biology and Drug Discovery, College of Medical Science and Technology, Taipei Medical University, Taipei 110, Taiwan; 9 Department of Pathology, Taipei Medical University Hospital and College of Medicine, Taipei Medical University, Taipei 110, Taiwan

**Keywords:** colorectal cancer, MAOB, prognosis, epithelial-to-mesenchymal transition

## Abstract

Monoamine oxidases (MAOs) including MAOA and MAOB are enzymes located on the outer membranes of mitochondria, which are responsible for catalyzing monoamine oxidation. Recently, increased level of MAOs were shown in several cancer types. However, possible roles of MAOs have not yet been elucidated in the progression and prognosis of colorectal carcinoma (CRC). We therefore analyzed the importance of MAOs in CRC by an in silico analysis and tissue microarrays. Several independent cohorts indicated that high expression of MAOB, but not MAOA, was correlated with a worse disease stage and poorer survival. In total, 203 colorectal adenocarcinoma cases underwent immunohistochemical staining of MAOs, and associations with clinicopathological parameters and patient outcomes were evaluated. We found that MAOB is highly expressed in CRC tissues compared to normal colorectal tissues, and its expression was significantly correlated with a higher recurrence rate and a poor prognosis. Moreover, according to the univariate and multivariate analyses, we found that MAOB could be an independent prognostic factor for overall survival and disease-free survival, and its prognostic value was better than T and N stage. Furthermore, significant positive and negative correlations of MAOB with mesenchymal-type and epithelial-type gene expressions were observed in CRC tissues. According to the highlighted characteristics of MAOB in CRC, MAOB can be used as a novel indicator to predict the progression and prognosis of CRC patients.

## 1. Introduction

Colorectal cancer (CRC) is one of the most common cancers among all races [1]. The incidence of CRC has rapidly increased in Asian countries during the last decade [2]. In 2017, CRC ranked at the top of malignant tumors in Taiwan according to incidence. The major therapeutic modality for CRC is surgery followed by chemotherapy in advanced tumors. However, tumor recurrence is found in approximately 30% of late-stage patients, and patients diagnosed at a late stage have a high mortality rate due to distant metastasis [3]. Although many aberrant genes and biomarkers have already been identified in CRC [4,5,6], there are still many challenges to overcome, ranging from the early diagnosis to the determination of prognosis factors and treatment of advanced disease, in order to establish a personalized approach. Therefore, improving the patients’ prognosis, the prediction of treatment response, and recurrence risk would be enabled with reliable biomarkers for early detection of CRC. At present, carcinoembryonic antigen (CEA) is the most commonly used blood-based biomarker for CRC in clinical practice, but it is limited by low sensitivity and specificity. Elevated CEA levels are associated with cancer progression and can indicate recurrence after surgery [7]. However, high CEA levels are not specific to CRC and can also be found in other inflammatory diseases, such as inflammatory bowel disease, pancreatitis, and other malignancies [8]. Carbohydrate antigen 19-9 (CA 19-9) is another biomarker available to monitor CRC patients, but its sensitivity and specificity for CRC is less than CEA [7]. In addition, microsatellite instability (MSI) is a molecular marker associated with defective DNA mismatch repair and can be detected in approximately 15% of sporadic colon cancers. At present, five microsatellite markers are used for MSI analysis, classifying the cancer as MSI-high (MSI-H), MSI-low (MSI-L), or MSI-stable (MSI-S) [9]. In clinical applications, MSI can provide valuable prognostic and predictive information in CRC patients. For example, CRC patients with MSI-H tumors have been reported to have better survival rates compared with those with non-MSI-H tumors [10]. Moreover, patients with MSI-H colon cancers display resistance to 5-Fluorouracil (5-FU)-based chemotherapy [10]. Despite these recent advances, the discovery of novel tumor diagnostic, predictive, or prognostic biomarkers is still urgently needed to optimize the management and follow-up of CRC patients.

Recently, several studies have discussed the expression levels of monoamine oxidase (MOA) A (MAOA) and MAOB in cancers; however, controversial characteristics were identified for these two enzymes. For example, downregulation of MAOA was found in several cancers, including malignant melanomas, breast cancer, and cholangiocarcinomas [11,12]. Downregulation of MAOA messenger (m)RNA levels was suggested as a biomarker for CRC [13], but neither the protein expression nor associations between MAOA and clinicopathological parameters in colorectal cancer have been addressed. In contrast, the oncogenic roles of MAOA in promoting the epithelial-to-mesenchymal transition (EMT) process were reported in prostate cancer [14,15]. Although upregulation and downregulation of MAOB were respectively observed in gliomas [16] and betel nut-associated oral cancer [17], the role and biologic significance of MAOB in pathogenesis were rarely independently mentioned in those studies. The predominant expression of MAOs is in the gastrointestinal tract, but detailed investigations of MAOs in CRC progression and prognosis remain limited. First, we surveyed mRNA expression levels of MAOA and MAOB in CRC tissues from three available online databases, Prediction of Clinical Outcome from Genomic Profiles (PRECOG), SurvExpress, and Gene Expression Omnibus (GEO), and also performed immunohistochemical (IHC) staining of MAOA and MAOB using CRC tissue microarrays (TMAs). Associations of MAOs with clinicopathological parameters and survival rates were further evaluated. Our results from TMAs showed that a high MAOB expression level was observed in CRC tissue and positively correlated with a higher recurrence rate and a poor prognosis in CRC patients. In silico analysis showed the positive correlations of MAOB and mesenchymal-type gene expressions in CRC tissues.

## 2. Results

### 2.1. Higher Expression of MAOB, but Not MAOA, Correlated with a Poorer Prognosis in CRC Patients

**Figure 1:**
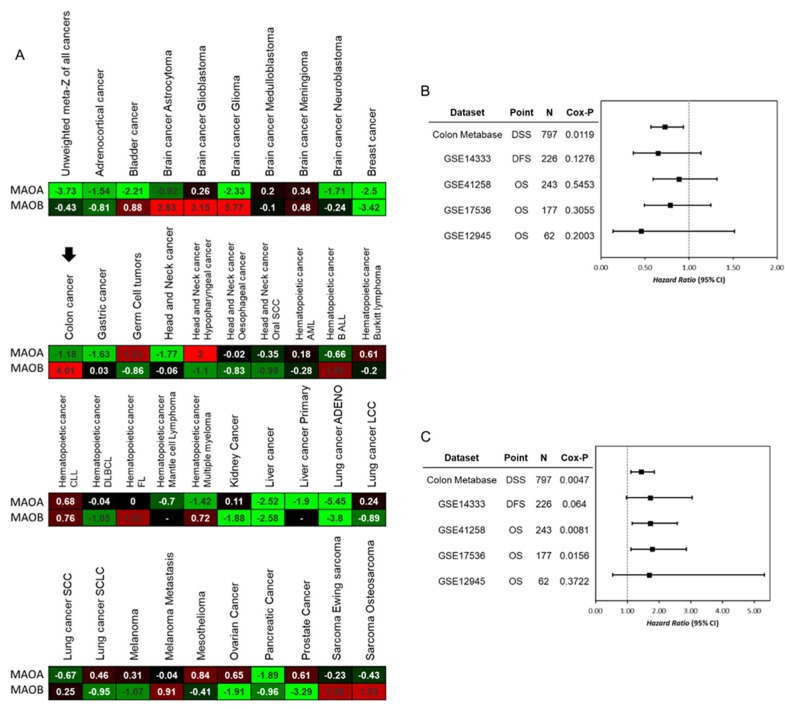
The clinical value of monoamine oxidase A (MAOA) and MAOB in colorectal cancer. (**A**) Visualization of the pan-cancer expression of MAOA and MAOB levels. Meta-Z analysis using the Prediction of Clinical Outcome from Genomic Profiles (PRECOG) website, which encompasses 39 distinct cancer types and 166 cancer expression datasets. (**B**,**C**) Forest plot showing the hazard ratios (HRs) and 95% confidence intervals for the association of MAOA (**B**) and MAOB (**C**) expression levels with disease-specific survival (DSS), disease-free survival (DFS), and overall survival (OS) in the five independent colorectal cancer (CRC) databases. The *p*-value was calculated.

Due to a limited understanding of the roles of MAOs in CRC, we first used the PRECOG [18] website (https://precog.stanford.edu/index.php) to evaluate mRNA expression levels of MAOs in colon cancer. Data showed significantly increased MAOB expression (Z score = 4.01) and slightly decreased MAOA expression (Z score = −1.18) in CRC tumor tissues (Figure 1A). In addition, we further evaluated the prognostic value of MAOs by an in silico analysis from several clinical cohorts in SurvExpress (http://bioinformatica.mty.itesm.mx/SurvExpress) [19]. The meta-analytical data indicated that higher expression of MAOA was not correlated with a poorer prognosis (Figure 1B). In contrast, elevated MAOB was significantly correlated with a worse prognosis in these cohorts (Figure 1C).

**Figure 2:**
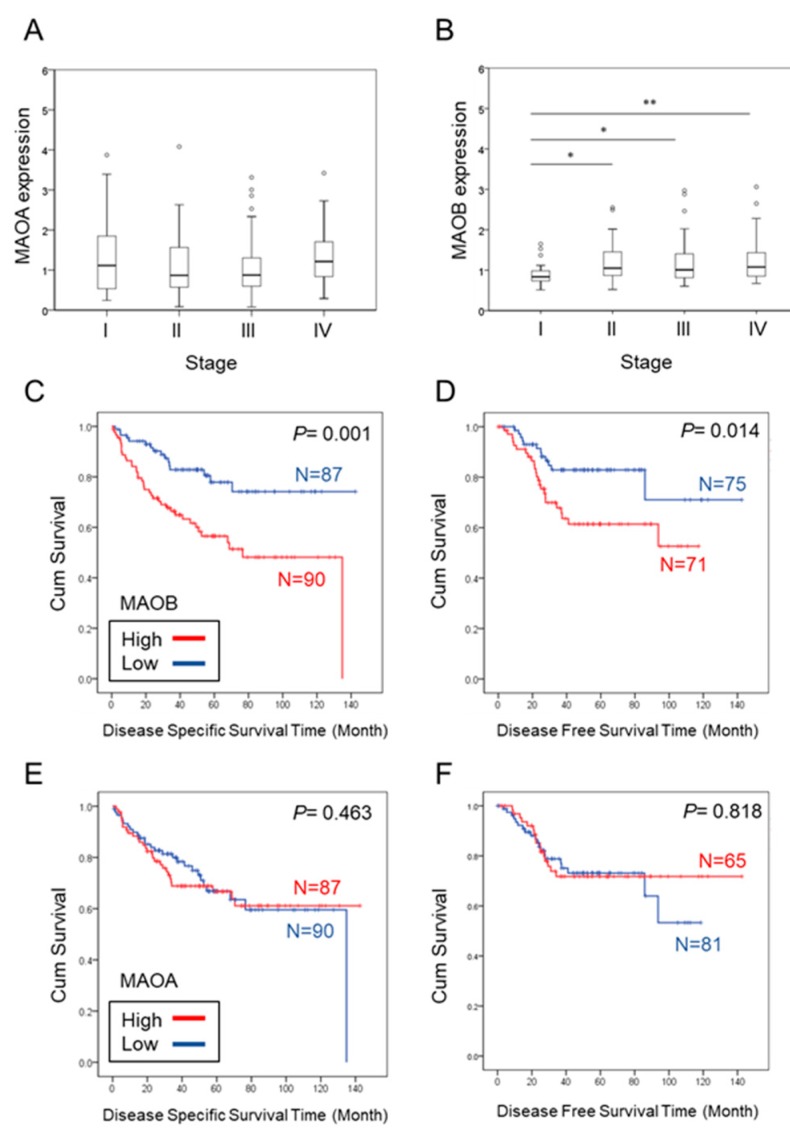
Correlation of the monoamine oxidase (MAO) A (MAOA) and MAOB expression levels with prognosis in a colorectal cancer database (GSE17536). (**A**,**B**) MAOA (**A**) and MAOB (**B**) gene expression levels in CRC tissues from the Gene Expression Omnibus (GEO; GSE17536) were compared according to clinical stages. The patient numbers of stage I, II, III, and IV were respectively 24, 57, 57, and 39. Statistical significance was analyzed by a *t*-test. * *p* < 0.05; ** *p* < 0.01. (**C**,**D**) Kaplan–Meier plots of the disease-specific survival (DSS) curves (**C**) and disease-free survival (DFS) curves (**D**) for MAOB (probe ID: 204041_at) expression. (**E**,**F**) Kaplan–Meier plots of DSS curves (**E**) and DFS curves (**F**) for MAOA (probe ID: 204388_s_at) expression. The average survival periods of DSS and DFS were 48.1 and 37.5 months, respectively. The red line indicates high expression of MAOs, and the blue line indicates low expression.

Next, we analyzed microarray data of GSE17536, which provided comprehensive clinicopathological data from 177 CRC patients. Messenger RNA (mRNA) expression levels of monoamine oxidase A (MAOA) in CRC of different stages were similar (Figure 2A). However, for monoamine oxidase B (MAOB), cancers in advanced stages (stages II, III, and IV) presented higher expression levels of MAOB than those in stage I (Figure 2B). In addition, we also analyzed correlations of MAOA and MAOB expressions with patient survival. Data showed that only high MAOB expression was strongly correlated with poor disease-specific survival (DSS; *p* = 0.001) and disease-free survival (DFS; *p* = 0.014) (Figure 2C,D). The MAOA expression level was not associated with these two survival probabilities (*p* = 0.463 and 0.818, respectively) (Figure 2E,F). According to these in silico analyses, higher MAOB, but not MAOA, expression might be a poor prognostic marker in CRC.

### 2.2. Immunohistochemical (IHC) Analysis of MAOA and MAOB in CRC Tissues

**Figure 3:**
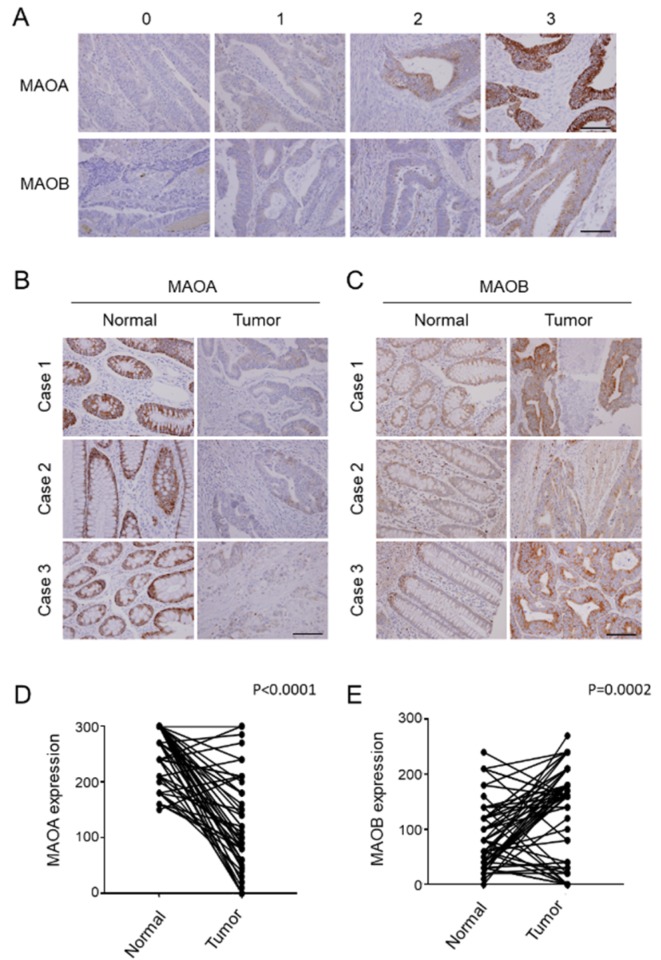
Immunohistochemical (IHC) results of monoamine oxidase A (MAOA) and MAOB expressions in a Taiwanese colorectal cancer cohort. (**A**) Representative pictures of expressions of MAOA and MAOB by IHC staining. An intensity score of 0 was defined as negative cytoplasmic staining, of 1 was defined as weak cytoplasmic staining, of 2 was defined as moderate cytoplasmic staining, and of 3 was defined as strong cytoplasmic staining. Scale bar indicated 100 μm. (**B**,**C**) Representative IHC staining images for MAOA (**B**) and MAOB (**C**) levels in paired normal (N) and tumor tissues (T) from selected colorectal cancer patients. The magnifying factor used in these representative pictures is ×400, and the intensity score of MAOA in the N part was 3. Scale bar indicated 100 μm. (**D**,**E**) Quantified results of cytoplasmic levels of MAOA (**D**) and MAOB (**E**) from IHC staining in primary colorectal cancer and corresponding normal colon mucosa. A total of 59 N/T paired data were included. The scores were calculated as the staining intensity score × percentage of stained cells.

To validate the observed correlations of MAOA and MAOB mRNA expressions with survival of CRC patients from the in silico analysis, IHC staining to determine MAOA and MAOB expression levels in CRC samples was performed. Representative examples of tumors showing overall negative (score 0), weak (score 1), moderate (score 2), and strong MAO (score 3) expressions are illustrated in Figure 3A. Among the 203 patients, 59 had paired normal colon tissues for comparing MAOA and MAOB expressions between non-tumor tissues and CRC tumor tissues. Our data showed that tumor tissues had significantly lower MAOA expression (*p* < 0.0001) and higher MAOB expression (*p* = 0.0002) compared to the paired non-tumor counterparts (Figure 3B,C). Generally, MAOA and MAOB were respectively downregulated and upregulated expressions in CRC tissues. 

### 2.3. Correlations between Expressions of MAOs and Clinicopathological Parameters of CRC Patients

Clinicopathological parameters of CRC patients are presented in Table 1. There were 203 patients, including 117 males and 86 females. Their ages ranged from 27 to 92 years old, with a mean value of 68.7 years. Of these patients, 108 cases (53.2%) had local recurrence or distant metastasis during the follow-up period. The overall average survival period of all cases was 58.6 (range, 1–146) months. The most common tumor location was the rectosigmoid colon (62.6%), followed by the ascending colon (17.2%), transverse colon (12.3%), and descending colon (7.9%). At diagnosis, 27 patients were at T1 or T2 stage, and 176 patients were T3 or T4 stage. Ninety-seven patients (47.8%) had no lymph node involvement. Most of the patients (89.2%) were at grade 2, with moderate differentiation. In total, 114 (54.2%) patients received adjuvant chemotherapy after the diagnosis. Of all 203 patients, 31 (15.3%) had distal metastasis at the time of the diagnosis, 110 (54.2%) had vascular invasion, and 46 (22.7%) had perineural invasion after pathologic observations. Fourteen cases (6.9%) were diagnosed as having a mucinous adenocarcinoma. Clinicopathological parameters, including age, sex, tumor location, T stage, N stage, M stage, vascular invasion, perineural invasion, tumor histology, and recurrence were used for the correlation analysis. According to MAO expression intensity and percentage scoring, high MAOA expression was found in 77 cases (37.9%) and high MAOB expression was observed in 114 cases (56.2%). MAOB expression levels were significantly correlated with recurrence (*p* = 0.0121). Other pathologic and clinical parameters had no correlation with MAO expressions.

### 2.4. Prognostic Value of MAO Expressions in a Taiwanese Colorectal Cancer Cohort

**Figure 4:**
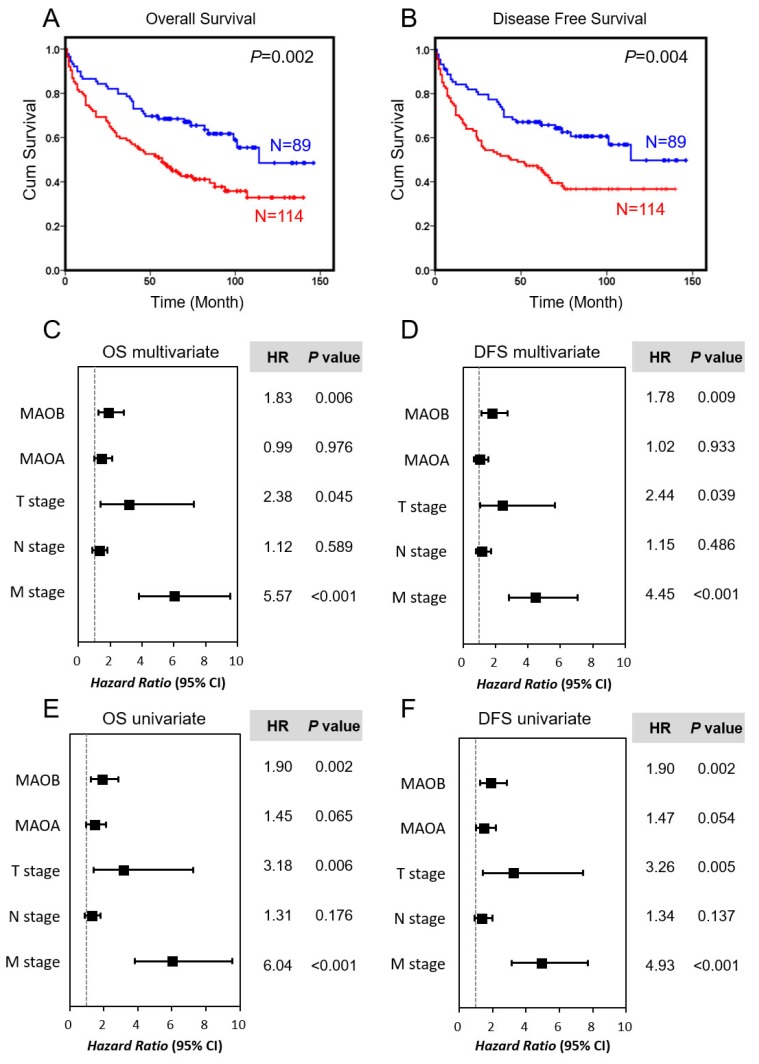
Prognostic value of monoamine oxidase B (MAOB) in colorectal cancer. (**A**,**B**) Kaplan–Meier plots of overall survival (OS) curves (**A**) and disease-free survival (DFS) curves (**B**) for MAOB in colorectal cancer patients. The red line indicates high expression of MAOB, and the blue line indicates low expression. (**C**,**D**) Forest plots of the multivariate analysis for hazard ratios (HRs) using a Cox regression model in OS (**C**) and DFS (**D**). (**E**,**F**) Forest plots of univariate analysis for HRs using a Cox regression model in OS (**E**) and DFS (**F**). The *p*-value was calculated.

MAO expression levels in 203 samples were also used to determine their correlation with patient survival by the Kaplan–Meier method and log-rank test (Figure 4). Overexpression of MAOB was significantly associated with a worse prognosis, both in terms of overall survival (OS; *p* = 0.002) (Figure 4A) and DFS (*p* = 0.004) (Figure 4B). The Cox proportional hazard model was also used to analyze hazard ratios (HRs) for relationships of clinicopathological parameters with OS and DFS. For OS (Figure 4C,E), higher MAOB expression, T stage, and M stage all shortened survival periods in both the univariate and multivariate analyses (HR = 1.9, 3.18, and 6.04, 95% confidence interval (CI) = 1.26–2.86, 1.40–7.27, and 3.81–9.56, *p* = 0.002, 0.006, and < 0.001 for the univariate analysis; HR = 1.83, 2.38, and 5.57, 95% CI = 1.19–2.82, 1.02–5.53, and 3.48–8.91, *p* = 0.006, 0.045, and <0.001 for the multivariate analysis, respectively). In the DFS panel (Figure 4D,F), results were similar to the OS analyses—higher MAOB expression, higher T stage, and a positive M stage independently and significantly affected survival periods of CRC patients (HR = 1.9, 3.26, and 4.93, 95% CI = 1.26–2.85, 1.42–7.43, and 3.14–7.72, *p* = 0.002, 0.005, and < 0.001 for the univariate analysis; HR = 1.78, 2.44, and 4.45, 95% CI = 1.15–2.74, 1.05–5.67, and 2.81–7.05, *p* = 0.009, 0.039, and <0.001 for the multivariate analysis, respectively). Importantly, the prognostic value of MAOB expression was better than that of the T or N stage in these colon cancer patients. We also stratified our recruited CRC patients into subgroups according to the pathological stage, and results indicated that high MAOB expression remained significantly correlated with a poor prognosis in patients with late stage (stage III + IV; *p* = 0.008 for OS and *p* = 0.024 for DFS) (Figure S1A, lower panel). Although a significant correlation between MAOB and poor prognosis was not observed in patients in the early stages (stage I + II; *p* = 0.275 for OS and *p* = 0.258 for DFS), the data still showed that these patients with higher MAOB levels had a trend of being associated with a poor prognosis (Figure S1A, upper panel). In addition, we also checked the prognostic role in different stages using the GEO dataset, GSE17536. Results also suggested that MAOB was associated with a poor prognosis, especially in stages III and IV (Figure S1B, lower panel).

### 2.5. Correlation between MAOB Expression Levels and EMT-Related Markers

**Figure 5:**
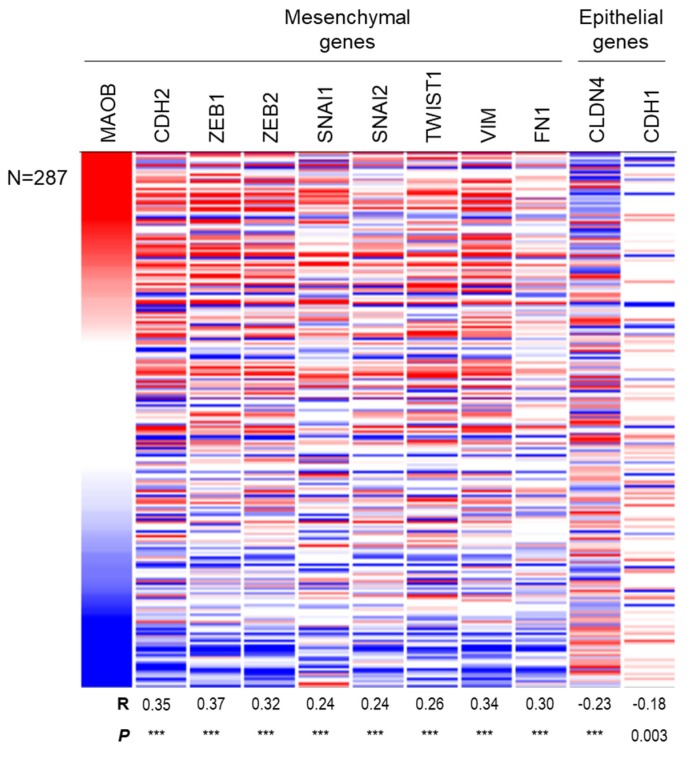
The correlation of MAOB expression with epithelial-to-mesenchymal transition (EMT)-related genes in colorectal cancer patients. Visualization of expressions of MAOB and EMT-related genes in 287 colon cancer patients from The Cancer Genome Atlas (TCGA) cohort. Positive correlations between MAOB and mesenchymal markers and negative correlations between MAOB and epithelial markers. R indicates the Pearson’s *R* value, and *** indicates *p* < 0.0001.

The EMT is one of the most important pathways to promote tumor progression including metastasis and growth in many cancer types, including breast, lung, and colon cancers [20,21,22]. Because a strong association between MAOB expression and a poor prognosis was observed, we therefore hypothesized a connection between MAOB and the EMT pathway in colon cancer. The meta-analysis of the expression of MAOB and mesenchymal gene expressions such as CDH2 (N-cadherin), ZEB1/2 (Zinc finger E-box-binding homeobox 1/2), SNAI1 (Snail), SNAI2 (Slug), TWIST1 (Twist-related protein 1), VIM (Vimentin), and FN1 (fibronectin-1) were further evaluated using The Cancer Genome Atlas (TCGA) database. The data revealed that MAOB exerted a strong and significant positive correlation with these mesenchymal markers in colon cancer samples (all *p-*values < 0.0001) (Figure 5). In contrast, negative correlations of MAOB and the epithelial type-related genes, CLDN4 (Claudin 4) and CDH1 (E-cadherin), were also observed in our analysis (Figure 5). In contrast to MAOB, MAOA was significantly correlated with epithelial markers, but was partially negatively correlated with mesenchymal markers (Figure S2). Mechanically, MAOB might promote colon cancer progression through inducing EMT progression.

## 3. Discussion

Clinical applications according to biomarker functions can be separated into diagnostic, predictive, or prognostic biomarkers. A diagnostic biomarker is defined as a characteristic to detect the presence of a disease or identify an individual with a subtype of the disease [23]. Our data found MAOB expression was significantly higher in CRC tissues compared to the non-tumor counterparts. However, MAOB expression was not restricted to CRC tissues and was not correlated specifically to the TNM stage of grade of CRC, suggesting the fact that MAOB is not suitable for a diagnostic biomarker in CRC. The predictive biomarkers were applied to indicate the response from a specific treatment and to guide the decision-making process. Recently, the increasing number of therapeutic drugs (chemotherapy, immunotherapy, and target therapy) used for CRC make it necessary to discover some response parameters and monitoring evaluations [24]. Unfortunately, we did not obtain detailed information about the treatment protocols for our recruited CRC patients, and thus it is worthy to further investigate whether MAOB can be a predictive biomarker for a specific treatment in CRC. In addition to diagnostic and predictive biomarkers, the prognostic biomarker is defined as a characteristic that offers information about the patient’s overall cancer outcome (OS, DFS, and risk of recurrence), independent of therapy [25]. Our data showed that CRC patients with high MAOB expression in the primary tumor had worse OS and DFS, as well as higher recurrence rate than patients with low MAOB expression. We defined the fact that MAOB can be an independent prognostic biomarker of OS and DFS in patients with CRC. The poor prognostic value of MAOB might be due to the regulation of EMT process by MAOB in CRC. In contrast, MAOA levels had no significant association with any clinicopathological parameters but had a potential correlation with sex, the N stage, and M stage in colon cancer. Due to the 70% similarity in their amino acid sequences, the *MAOA* and *MAOB* genes have similar functions in cells [26]. However, our results showed that the expression levels of these two proteins in CRC are opposite. The upstream regulation of these two genes in CRC is worthy of further evaluation.

The primary biological functions of both MAOA and MAOB are in the metabolism of neurotransmitters, and both enzymes can produce hydrogen peroxide through an oxidative reaction [27]. Additionally, overactivation of MAOs may increase oxidative stress and subsequent chronic inflammation, which ultimately may lead to many chronic diseases including cancer and neurological and pulmonary diseases. Oxidative stress can induce expressions of several transcription factors, including nuclear factor (NF)-κB, activating protein (AP)-1, hypoxia-inducible factor (HIF)-1α, β-catenin/Wnt, and nuclear respiratory factor (Nrf), which can subsequently stimulate the transformation of normal cells [28]. Recently, several studies reported that upregulation of MAOA could promote the EMT through the accumulation of oxidative stress and induction of hypoxia in prostate cancer cells [14]. However, whether MAOB can induce oxidative stress and promote the EMT in cancers such as CRC is still unclear. Our study, for the first time, revealed associations of MAOB and the EMT process in CRC.

Elevation of MAOB activity in cells led to induction of oxidative stress in association with mitochondrial dysfunction. Siddiqui et al. demonstrated that elevated MAOB was correlated with the aging process and Parkinsonian disease [29]. Our findings showed similar results, such as that elderly people presented with higher MAOB expression (with a threshold for the elderly at 65 years), and MAOB expression was correlated with disease progression in CRC. The dysfunction of mitochondria in cells would also force cells to use the glycolysis pathway to obtain ATP energy, which fits with the Warburg effect model in cancer cells. Thus, MAOB upregulation potentially drives cells metabolically closer to cancer cells. The possible mechanism involved in this phenomenon is not yet fully elucidated. In addition, MAOB expression was also found to be higher in the C85 colon cancer cell line treated with methotrexate, an anti-folate drug. Folate is an essential cofactor involved in DNA synthesis, repair, and methylation. MAOB might play an important role in regulating such DNA metabolic reactions [30].

Our current study is not without limitations. First, in our study, we did not record whether or not patients in our recruited cohort received radiation therapy, and thus we could not provide a correlation between MAOB and radiation therapy. Moreover, the patient number of our current study was still not very large, and this might have resulted in a limitation on the representativeness of the statistical results. For example, the rather small sample size of early stage I and II patients restricted the statistical relevance of MAOB in the early stages. Moreover, the relatively small sample size in our recruited cohort might also influence the prognostic significance of N stage that had been reported as an independent prognostic factor for OS and DFS in patients with CRC [31,32]. Our current data showed that the N stage only slightly but not significantly correlated with the higher hazard ratio in disease prognosis. Hence, future studies of enlarged patient cohort are necessary to confirm our results. Furthermore, the different criteria of patient collection from several online available datasets might have led to a limitation of the comprehensiveness of pathological characteristics in different cohorts. In these cohorts, we only evaluated the MAOB level and did not consider other pathological parameters. That might have restricted our observation to the level of MAOB and prognostic outcomes but not an integrated view of this complex malignant disease. Although we observed the fact that the MAOB gene exerted a strong correlation with mesenchymal markers in colon cancer samples from the TCGA database, we did not include the EMT-related markers as our validated genes when we applied for IRB (Institutional Review Board) approval at the beginning of this study. Therefore, we cannot further check the correlation of MAOB and mesenchymal markers by IHC stain in our current study, and thus this critical issue will be further investigated in our future work.

Altogether, MAOs may be involved in signaling regulation and many different metabolic reactions in cancer cells. Our data first showed that MAOA and MAOB expressions presented opposing regulatory impacts on CRC, as well as the fact that MAOB overexpression was associated with a poor prognosis in CRC. Our study presents a potential correlation between the metabolism and oxidation of monoamines in mitochondria and cancer progression through promoting the EMT process. However, whether MAOB is a driver of carcinogenesis in CRC has not been defined. Further molecular studies are necessary to elucidate the regulatory mechanisms of MAOB in CRC progression.

## 4. Materials and Methods 

### 4.1. Patients and Case Selection

In total, data on 203 CRC patients from Taipei Municipal Wan Fang Hospital in Taiwan, from 1998 to 2005, were collected for this study. Patients who received incomplete surgical resection, preoperative chemotherapy, or radiation therapy were excluded from the study. Clinical information and pathology data were collected via a retrospective review of medical records of these patients. The tumor, node, and metastasis (TNM) stage of each patient was determined according to the seventh edition of the Cancer Staging Manual of the American Joint Committee on Cancer, and histopathological types were classified according to World Health Organization classification. All therapeutic modalities of these patients were administered according to standard institutional treatment protocols. Patients received surgical resection, and patients at high risk underwent adjuvant chemotherapy including either 5-fluorouracil/leucovorin, capecitabine/oxaliplatin, doxorubicin/cisplatin/cyclophosphamide, or tegafur-uracil plus leucovorin. Follow-up data were recorded until January 2011, and patients were followed up for up to 146 months with a medium follow-up time of 60 months in this study. Overall survival (OS) was defined as the period from diagnosis to death from any cause, and disease-free survival (DFS) was defined as the period from diagnosis to cancer recurrence, distant metastasis, or death of the patient. The study was carried out with the approval of the Institutional Review Board of Wan Fang Hospital (approval no. 99047; approved on 4 November 2010) and permission from the ethics committees of the institution involved.

### 4.2. Tissue Microarray (TMA) Construction and IHC Staining

Three representative cores (1 mm) of paraffin-embedded tumor tissue of each case were arranged sequentially in a TMA. The dig fields were diagnostic with typical morphology as identified by a pathologist. Paired normal colon tissues were also obtained from 59 patients in this cohort. The adequacy of all samples in the TMA was confirmed by three pathologists (C.Y. Su, C.L. Chen, and M. Hsiao) via hematoxylin and eosin (H&E)-stained sections. IHC staining was performed using an automated immunostainer (Ventana Discovery XT autostainer, Ventana Medical Systems, AZ, USA). Slides were stained with polyclonal rabbit anti-human MAOA (GTX101289, GeneTex, Hsinchu, Taiwan) and MAOB antibodies (GTX105970, GeneTex). Only the cytoplasmic expression of tumor cells was evaluated. Both the staining intensity and the percentage of stained tumor cells were recorded. The intensity of staining was scored by the following definition: 0, no staining; 1+, weak staining; 2+, moderate staining; and 3+, strong staining. The purpose of the scoring was to calculate the percentage of positive cells (0–100%). The final IHC scores (0–300) were multiplied by the staining intensity score and the percentage of positive cells. All patients were divided into either a high- or low-expression group according to the IHC score by using a cutoff value of 150.

### 4.3. In Silico Analysis

Survival Z analysis data of MAOA and MAOB in different cancer subtypes were visualized according to the PRECOG (PREdiction of Clinical Outcomes from Genomic profiles) website (https://precog.stanford.edu/) [18]. Indicated survival Z scores reflect the relationship of each gene and the statistical significance of clinical prognosis outcomes, with red-colored blocks representing a poor prognosis and green blocks representing a favorable prognosis. Hazard ratios (HRs) of these two genes were obtained from the Survexpress website (http://bioinformatica.mty.itesm.mx/SurvExpress) [19]. GSE17536 microarray datasets were collected from the Gene Expression Omnibus (GEO) database (https://www.ncbi.nlm.nih.gov/geo/query/acc.cgi?acc=GSE17536) [33]. The series contains gene expression profiles of 177 colon cancer samples that provided the complete status of stage, grade, OS, disease-specific survival (DSS), and DFS. Raw data were normalized using a standard tissue microarray (TMA) method by GeneSpring (Agilent Technologies, Santa Clara, CA, USA). The probe of MAOA was 204388_s_at, and that of MAOB was 204041_at. We used the medium level of gene expression as the cutoff for separating high- and low-expression groups. About the correlations between MAOB and EMT-related markers, these gene expression levels were obtained and analyzed from the online available TCGA colon cancer cohort. The gene level transcription had been estimated as log2(normalized_count+1) and downloaded from “UCSC Xena Browser” website (https://xenabrowser.net/).

### 4.4. Statistical Analysis

Statistical analyses were performed with SPSS 20.0 software (IBM, Endicott, NY, USA). A paired *t*-test was used to compare IHC expressions between individual normal tissues and cancer tissues. Correlations between clinicopathological parameters and MAO gene expressions were assessed by Pearson’s chi-squared test. DFS and OS were analyzed by the Kaplan–Meier method and compared using a log-rank test. Univariate and multivariate analyses were performed by a Cox proportional hazard analysis with and without adjustment for protein expressions and various clinicopathological parameters. In all analyses, a *p*-value < 0.05 was considered statistically significant.

## Figures and Tables

**Table 1:** Correlations of clinicopathological features of colorectal cancer patients and tumor expressions of monoamine oxidase A (MAOA) and MAOB.

Clinicopathological Feature	*n*	MAOA Expression, *n* (%)		*p*	MAOB Expression, *n* (%)		*p-*Value
	203	Low	High		Low	High	
		126 (62.1)	77 (37.9)		89 (43.8)	114 (56.2)	
Age (years)							
<65	64	44 (68.8)	20 (31.2)	0.2401	34 (53.1)	30 (46.9)	0.0979
≥65	139	82 (59.0)	57 (41.0)		55 (40.0)	84 (60.0)	
Gender							
Male	117	66 (56.4)	51 (43.6)	0.0732	45 (38.5)	72 (61.5)	0.0973
Female	86	60 (69.8)	26 (30.2)		44 (51.2)	42 (48.8)	
Tumor location							
Right colon	35	20 (57.1)	15 (42.9)	0.7988	10 (28.6)	25 (71.4)	0.2519
Transverse colon	25	17 (68.0)	8 (32.0)		12 (48.0)	13 (52.0)	
Descending colon	16	9 (56.3)	7 (43.7)		7 (43.8)	9 (56.2)	
Rectosigmoid colon	127	80 (63.0)	47 (37.0)		60 (47.2)	67 (52.8)	
T stage							
T1+T2	27	17 (63.0)	10 (37.0)	0.9203	14 (51.9)	13 (48.1)	0.4884
T3+T4	176	109 (61.9)	67 (38.1)		75 (42.6)	101 (57.4)	
N stage							
N0	97	64 (66.0)	33 (34.0)	0.0633	48 (49.5)	49 (50.5)	0.1594
N1+N2	106	62 (58.5)	44 (51.5)		41 (38.7)	65 (61.3)	
M stage							
M0	172	112 (65.1)	60 (34.9)	0.0564	78 (45.3)	94 (54.7)	0.4096
M1	31	14 (45.2)	17 (54.8)		11 (35.5)	20 (64.5)	
TNM stage							
Stage I	21	15 (71.4)	6 (28.6)	0.1089	12 (57.1)	9 (42.9)	0.1208
Stage II	65	45 (69.2)	20 (30.8)		34 (52.3)	31 (47.7)	
Stage III	86	52 (60.5)	34 (39.5)		32 (37.2)	54 (62.8)	
Stage IV	31	14 (45.2)	17 (54.8)		11 (35.5)	20 (64.5)	
Grade							
Grade 1	4	3 (75.0)	1 (25.0)	0.6883	3 (75.0)	1 (25.0)	0.3743
Grade 2	181	112 (61.9)	69 (38.1)		76 (42.0)	105 (58.0)	
Grade 3	8	6 (75.0)	2 (25.0)		5 (62.5)	3 (37.5)	
NA	10	5 (50.0)	5 (50.0)		5 (50.0)	5 (50.0)	
Adjuvant chemotherapy							
No	89	58 (65.2)	31 (34.8)	0.4213	40 (44.9)	49 (55.1)	0.7799
Yes	114	68 (59.6)	46 (40.4)		49 (43.0)	65 (57.0)	
Vascular invasion							
No	93	56 (60.2)	37 (39.8)	0.7184	42 (45.2)	51 (54.8)	0.8415
Yes	110	70 (63.6)	40 (36.4)		47 (42.7)	63 (57.3)	
Perineural invasion							
No	157	94 (60.0)	63 (40.0)	0.3078	70 (44.6)	87 (55.4)	0.8231
Yes	46	32 (69.6)	14 (30.4)		19 (41.3)	27 (58.7)	
Tumor histology							
Non-mucinous	189	118 (62.4)	71 (37.6)	0.9203	83 (43.9)	106 (56.1)	0.8415
Mucinous	14	8 (57.1)	6 (32.9)		6 (42.9)	8 (57.1)	
Recurrence							
No	95	63 (66.3)	32 (33.7)	0.3055	51 (53.7)	44 (47.3)	0.0121
Yes	108	63 (58.3)	45 (41.7)		38 (35.2)	70 (64.8)	

TNM, tumor, node, metastasis; NA, not applicable.

## Supplementary Materials

Supplementary materials can be found at https://www.mdpi.com/1422-0067/21/8/2813/s1.

## Abbreviations

CRC	Colorectal cancer
DFS	Disease-free survival
DSS	Disease-specific survival
EMT	Epithelial-to-mesenchymal transition
IHC	Immunohistochemical
MAO	Monoamine oxidase
OS	Overall survival
TCGA	The Cancer Genome Atlas
TMA	Tissue microarray
