# Supplementary material for: Monoamine Oxidase B Expression Correlates with a Poor Prognosis in Colorectal Cancer Patients and Is Significantly Associated with Epithelial-to-Mesenchymal Transition-Related Gene Signatures
Source: Int J Mol Sci. 2020 Apr 17;21(8):2813. doi: 10.3390/ijms21082813 (PMC7215409; doi:10.3390/ijms21082813)
Supplement: Supplementary file 1 [file ijms-21-02813-s001.pdf]

Monoamine Oxidase B Expression Correlates with a Poor Prognosis in Colorectal Cancer Patients and Is Significantly Associated with Epithelial-to-Mesenchymal Transition-related Gene Signatures

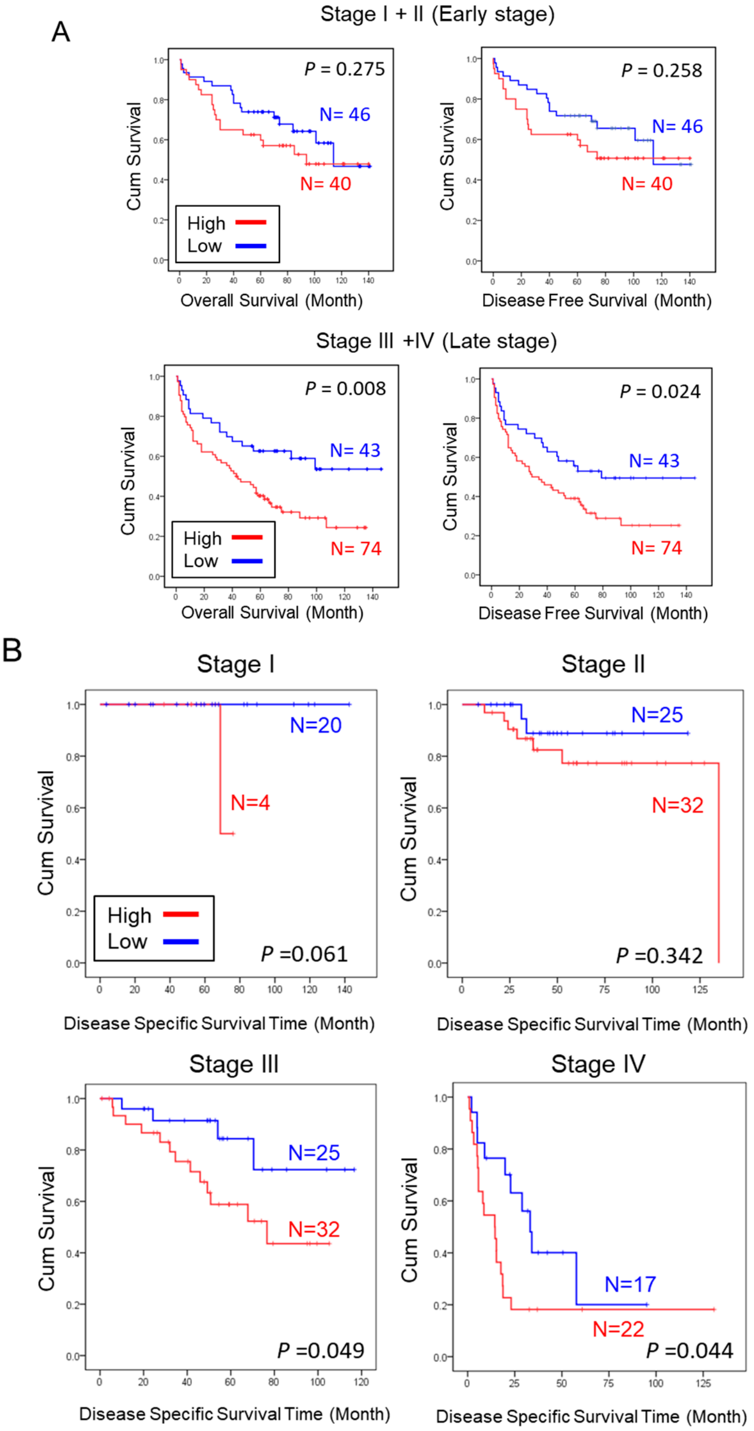

Supplementary Figure S1. (A) Kaplan-Meier plots of overall survival and disease-free survival curves for monoamine oxidase B (MAOB) protein levels in Taiwanese colorectal cancer patients in early stages (stages I + II) and late stages (stages III + IV). (B) Kaplan-Meier plots of disease-specific survival curves for MAOB mRNA levels in the indicated stages from the GEO dataset, GSE17536.

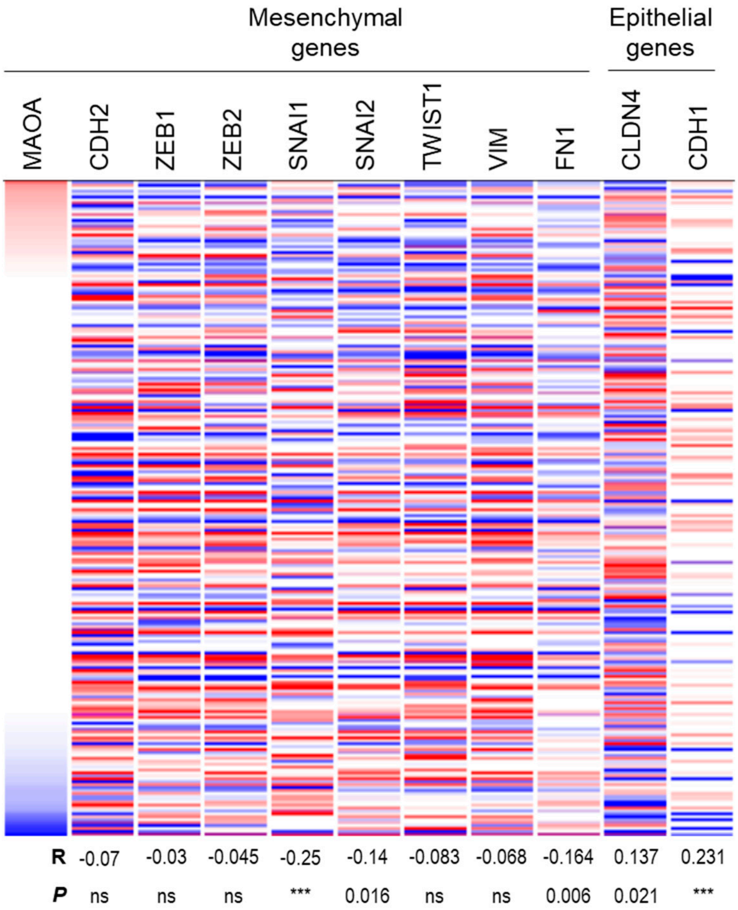

Supplementary Figure S2. Correlations of monoamine oxidase A (MAOA) expression with epithelial-to-mesenchymal transition (EMT)-related genes in colorectal cancer patients. Visualization of the expressions of MAOA and EMT-related genes in 287 colon cancer patients from TCGA cohort. Negative correlations between MAOA and some mesenchymal markers and positive correlations between MAOA and epithelial markers. ***R*** indicates the Pearson R value, and *** indicates $p < 0.0001$. ns: not significant.
